# Investigating a Novel Activation-Repolarisation Time Metric to Predict Localised Vulnerability to Reentry Using Computational Modelling

**DOI:** 10.1371/journal.pone.0149342

**Published:** 2016-03-02

**Authors:** Yolanda R. Hill, Nick Child, Ben Hanson, Mikael Wallman, Ruben Coronel, Gernot Plank, Christopher A. Rinaldi, Jaswinder Gill, Nicolas P. Smith, Peter Taggart, Martin J. Bishop

**Affiliations:** 1 Department of Biomedical Engineering, Division of Imaging Sciences & Biomedical Engineering, King’s College London, London, United Kingdom; 2 Department of Mechanical Engineering, University College London, London, United Kingdom; 3 Department of Systems and Data Analysis, Franhofer-Chalmers Centre, Gothenburg, Sweden; 4 Academic Medical Center, Amsterdam, Netherlands; 5 L’Institut de RYthmologieet de Modelisation Cardiaque (LIRYC), Fondation Universite Bordeaux, Bordeaux, France; 6 Institute of Biophysics, Medical University of Graz, Graz, Austria; 7 Department of Cardiology, Guys and St Thomas’ Hospital, London, United Kingdom; 8 Department of Engineering, University of Auckland, Auckland, New Zealand; 9 Department of Cardiovascular Sciences, University College London, London, United Kingdom; Gent University, BELGIUM

## Abstract

Exit sites associated with scar-related reentrant arrhythmias represent important targets for catheter ablation therapy. However, their accurate location in a safe and robust manner remains a significant clinical challenge. We recently proposed a novel quantitative metric (termed the Reentry Vulnerability Index, RVI) to determine the difference between activation and repolarisation intervals measured from pairs of spatial locations during premature stimulation to accurately locate the critical site of reentry formation. In the clinic, the method showed potential to identify regions of low RVI corresponding to areas vulnerable to reentry, subsequently identified as ventricular tachycardia (VT) circuit exit sites. Here, we perform an *in silico* investigation of the RVI metric in order to aid the acquisition and interpretation of RVI maps and optimise its future usage within the clinic. Within idealised 2D sheet models we show that the RVI produces lower values under correspondingly more arrhythmogenic conditions, with even low resolution (8 mm electrode separation) recordings still able to locate vulnerable regions. When applied to models of infarct scars, the surface RVI maps successfully identified exit sites of the reentrant circuit, even in scenarios where the scar was wholly intramural. Within highly complex infarct scar anatomies with multiple reentrant pathways, the identified exit sites were dependent upon the specific pacing location used to compute the endocardial RVI maps. However, simulated ablation of these sites successfully prevented the reentry re-initiation. We conclude that endocardial surface RVI maps are able to successfully locate regions vulnerable to reentry corresponding to critical exit sites during sustained scar-related VT. The method is robust against highly complex and intramural scar anatomies and low resolution clinical data acquisition. Optimal location of all relevant sites requires RVI maps to be computed from multiple pacing locations.

## Introduction

Ischemic heart disease remains a significant cause of world-wide mortality and morbidity, primarily due to ventricular arrhythmia. Areas of localised myocardial scars support ventricular tachycardia (VT) circuits by providing a substrate around which reentrant waves form [1]. Radio-frequency catheter ablation is a widely-used clinical therapy [2] aimed at interrupting the reentrant pathway associated with the scar by creating lesions, thus terminating and preventing reentry. However, success rates of ablation are low, particularly in cases of scar and cardiomyopathy where they may be as low as ∼50% [3] for VT re-occurrence. It therefore remains a significant challenge to successfully and safely locate the optimal target for ablation.

Infarct scars usually comprise of regions of non-conducting, dense, fibrotic tissue surrounded by remodelled [4], yet still viable, myocardium, which constitutes the infarct border-zone (BZ). Channels, or isthmuses, of surviving tracts of myocardium interspersed with fibrosis [5] may also exist through an otherwise dense scar. Such channels provide a slow, yet viable, conduit for activation to propagate due to the tortuous pathways the wavefront is forced to take [6]. During reentry, the resulting conduction delay [7] allows the tissue at the exit point of the isthmus to repolarise, such that it may be reactivated by these anomalous waves, providing the necessary requirement for self-sustained reentrant activity. Such mechanisms underlie the majority of incidences of scar-related VT.

The conduction delay introduced by the prolonged activation pathway within the isthmuses plays a key role in the scar’s tendency to act as a strong arrhythmic substrate. Accurate identification of the entry/exit points of the isthmuses therefore provide an important target for catheter ablation therapy. In order to accurately identify exit sites, it is often necessary to induce VT in the electrophysiology (EP)-lab, increasing the risk of the procedure as the VT may not be haemodynamically tolerated. The VT may also be difficult to induce and sustain, and may be mechanistically different to spontaneously occurring clinical VT. In cases where VT is not tolerated or inducible, voltage-mapping or detection of fractionated electrograms may be used to locate isthmus regions; however, in these cases it is harder to identify the critical exit sites forming part of the reentrant circuit associated with the VT. Additionally, multiple circuits and exit points may be present [8] which are often initially missed, leading to recurrence of VT at a later stage. Alternatively, in an attempt to ablate all possible exit sites, the ablation scar may span large regions which were not necessary to ablate, potentially leading to mechanical dysfunction [9].

Recent work from our group [10] has attempted to address many of these issues by developing a novel metric (termed the Reentry Vulnerability Index (RVI)) to accurately locate multiple localised regions of tissue vulnerable to reentry. The RVI is derived from spatial activation and repolarisation time maps obtained during a pacing protocol and thus does not require the need for VT-induction. Adoption of this approach therefore potentially provides a valuable means of more safely and robustly guiding catheter ablation within the clinic. The RVI metric is based on an algorithm derived in a previous experimental study [11, 12] which quantitatively assessed the likelihood of excitation wavefront-waveback interactions either side of a line of conduction block occurring proximal to a region of inexcitability following a premature stimulus. The algorithm effectively assesses whether the wavelength of the excitation is greater than the physical (reentrant) path length between two points spanning the line of block, thus assessing its vulnerability to sustain a stable reentrant circuit. Child et al [10] developed this idea to produce a spatial map of the RVI metric over the endocardial surface of the heart in a patient undergoing catheter ablation for recurrent scar-related VT, computed using only decapolar catheter recordings of activation times (AT) and repolarisation times (RT) following S1S2 pacing. They demonstrated that the method showed the potential to identify sites of low RVI corresponding to critical sites of vulnerability to reentry identified as VT circuit exit sites following subsequent induction.

Many questions remain regarding the use of the RVI mapping algorithm in clinical practice to ensure the optimal application and robustness of the approach under the widely varying physiological scenarios occurring throughout the patient population. The goal of this study is to use a series of computational models to mechanistically understand and advance the use of the RVI algorithm within clinical ablation procedures by optimising the acquisition, processing and interpretation of the clinical data. Use of a multi-scale *in silico* approach provides detailed three-dimensional electrophysiological information to facilitate analysis of the RVI metric in the context of variations in both structural and functional properties which may be more easily altered in the models relative to experimental or clinical investigations. Firstly, a highly-simplified two-dimensional model is used, based on the previously described experimental set-up [11, 12], to provide a highly controlled environment in order to gain an in-depth understanding of how parameters affecting the calculation of the RVI may be optimised, as well as how the numerical value and spatial distribution of the RVI metric is altered under increasingly arrhythmogenic conditions. An idealised representation of an arrhythmogenic scar substrate is then incorporated within a simplified cuboid model to critically understand the behaviour of the RVI metric in the context of reentrant pathways associated with an infarct scar, and its ability to identify situations of intramural reentry. Finally, in order to investigate the affects of different pacing locations with respect to different scar anatomies on the success of the RVI approach, an anatomically-detailed whole ventricle model is used with a variety of realistic representations of infarct scar with conducting isthmus and BZ.

## Methods

### Geometrical Models

Three separate geometrical finite element models were employed in this study: a 2D sheet with no structural obstacles, a regular 3D cuboid incorporating an idealised representation of an infarct scar and BZ, and finally an anatomically-detailed rabbit whole ventricular model including an anatomically accurate representation of infarct scar anatomy and BZ.

#### 2D Structurally-Homogeneous Sheet

The 2D sheet consisted of a square 5 × 5 cm finite element mesh with nodal spacing of 200 *μ*m. Linear quadrilateral finite elements were used, with a total of 62,500 elements. Ionic membrane dynamics were represented using the Ten Tusscher human ventricular cell model [13]. As in the original experiment setups [10, 11], an action potential duration (APD) gradient was imposed between the upper and lower halves of the tissue by assigning different values to the maximum conductivity of the slow delayed rectifying potassium current (*I*_*Ks*_): *g*_*Ks*_ = 0.75 nS/pF in the upper half (proximal tissue), whilst *g*_*Ks*_ = 0.2 nS/pF in the lower half (distal tissue), giving respective APDs of ∼250 ms and ∼300 ms in the two regions. Isotropic tissue conductivity was assigned to the tissue with a value of 0.1 S/m.

#### 3D Cuboid with Idealised Scar

The 3D cuboid consisted of a 2 × 2 × 1 cm finite element mesh, also with nodal spacing of 200 *μ*m and ionic membrane dynamics represented by the same human ventricular cell model as above. Linear quadrilateral finite elements were used, with a total of 500,000 elements. This model included an idealised representation of an infarct scar, consisting of two ellipsoidal necrotic regions, separated by a conducting isthmus. The axes of the ellipsoids were 8 mm, 4 mm and 5 mm in the *x*−, *y*− and *z*− directions, respectively, with their centres a distance 7 mm apart along the *y*-direction. The centres of the scar regions were initially located within the plane of the upper surface (*z* = 0 mm) as default. The tissue within the isthmus through the scar was set to be BZ tissue with ionic cell properties adjusted based on [14, 15] to produce a lengthened APD in this region. Specifically, the conductance of the rapid delayed rectifier potassium current (*g*_*Kr*_) was reduced to 20%, the conductance of the slow delayed rectifier potassium current (*g*_*Ks*_) was reduced to 30% and the conductance of the sodium current was reduced to 38%. APD was correspondingly lengthened from a value of ∼250 ms within normal myocardium to ∼330 ms in BZ tissue. Tissue conductivity in the model was set to be isotropic with a value of 0.05 S/cm, ensuring a reentrant circuit could fit within the relatively small domain. Within the BZ tissue, conductivity was further reduced in order to reduce CV by 45%, Such a representation had the desired effect of replicating the known conduction delay due to tortuous, zig-zag activation patterns taken by activation within this region [5, 6].

#### Whole Ventricular Scar Anatomy Models

**Fig 1:**
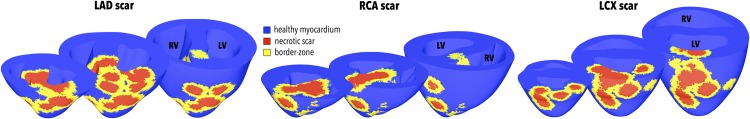
Three different anatomically-based representations of infarct scar and BZ within the rabbit ventricular model based on infarct formation following occlusion of the LAD, RCA and LCX. Healthy ventricular tissue is shown in blue with necrotic scar in red and BZ tissue in yellow. For each individual scar anatomy, three separate short-axis clipping plane views (near the apex, mid- and base) have been used to highlight intramural scar anatomy.

The histologically-based whole ventricular rabbit model consisted of a tetrahedral finite element model, as described in previous studies [16, 17]. Linear tetrahedral elements were used, with a total of 3,074,982 elements. Within this ventricular model, an anatomically-detailed region of infarct scar and corresponding BZ was assigned using a previously published algorithm [18]. Briefly, the algorithm consists of the following steps: 1) Definition of the volume supplied by the relevant artery; 2) Random placement of 7–17 seed points within the area; 3) Computation of the minimal distance, *d*, from any seed point to each node in the mesh (using *Dijkstra’s algorithm*); 4) Thresholding of *d* to define scar (*d* < 5000 *μ*m) and BZ (5000 ≤ *d* < 7000 *μ*m). The utility of such an approach allowed different scar anatomies to be assigned to the same geometric ventricular model, facilitating direct comparison. Three different scar anatomies were defined representing approximate infarct scar regions created following occlusion of the left anterior descending (LAD), the right coronary artery (RCA) and the left circumflex (LCX) by assigning the initial ‘seed points’ for the scars to be within the respective perfusion regions of these vessels. Parameters of the scar creation algorithm were specifically chosen to produce complex scar/ BZ anatomies with a number of isthmuses and potential exit sites, characteristic of those complex scars seen in patients presenting with scar-related VT in the clinic. Fig 1 shows the three different scar anatomies created.

Ionic dynamics within the rabbit ventricular model were represented by a recent rabbit ventricular cell model [19]. Similar modifications to ionic currents as above were made within BZ regions based on previous studies [15] to produce a prolonged APD of ∼ 210 compared to healthy myocardium (∼160 ms). Such specific values were used to reproduce the required region of prolonged APD (to facilitate the initial uni-directional block) with slow, yet viable, conduction (to facilitate the accommodation of a reentrant circuit). The model contained realistic representation of fibre architecture derived from histological information, as described in previous studies [16, 20]. Bulk conductivities along the fibre and cross-fibre directions were based on those of Clerc et al [21], reduced so as to slow CV by ∼25%, replicating the experimental use of flecainide to slow conduction [22], due to the known difficulties in successfully sustaining reentrant activity in healthy rabbit ventricles [23–25]. Within BZ regions, conduction was slowed further by ∼25%, again replicating the known conduction delay through these pathological regions [5, 6].

### Electrophysiological Calculations

Electrophysiological activity was simulated within the models using the Cardiac Arrhythmia Research Package (CARP) [26] (http://carp.meduni-graz.at). Throughout, a monodomain representation of tissue electrical dynamics was used with ionic dynamics represented by the above described cell models for each specific geometrical model used.

### RVI Calculation

The RVI algorithm, originally proposed in [11, 12], quantitatively assesses the likelihood of wavefront and waveback interactions around a reentrant circuit. If cardiac tissue is stimulated prematurely, conduction block may occur in regions with prolonged refractoriness (such as BZ of scar tissue). In such cases, initial uni-directional block occurs and the wavefront is forced around the line of block, attempting to reenter the proximal tissue (from where it originated) from the distal side of the line of block. If the proximal tissue has successfully repolarised such that it may be excited once again, reentry ensues; however, if the proximal tissue remains refractory, the wavefront is blocked from reentering across the line of initial block. Thus, for two neighbouring sites spanning the line of block, quantitatively comparing the activation time (AT) of the distal tissue and the repolarisation time (RT) of the proximal tissue has been closely correlated with reentry success [10–12]. It is this fundamental idea which is used as the basis for the RVI algorithm.

As described in Child *et al* [10], the RVI pacing protocol involves delivery of an S1 steady-state pacing train, followed by a premature S2 stimulus delivered at the same location. ATs and RTs are then derived for all recording sites (in this case each finite element node in the respective models) for the S2 beat. Briefly, AT was defined as the time at which *V*_*m*_ crossed −20 mV (with positive gradient), whilst RT was defined as the time following activation at which *V*_*m*_ crossed −70 mV with (negative gradient). The resulting spatial AT and RT maps generated are then used in the computation of the RVI, which involves comparing differences in AT and RT between pairs of recording sites. Briefly, for a given recording site (*i*) all other sites (*j*) that are activated later than site *i* (i.e. are downstream of it) and lie within a given search radius (*r*_*s*_) are found. The RVI for this recording site pair (*RV I*_*ij*_) is then computed as

$$RV\;I_{ij}=RT_{i}-AT_{j},$$ (1)

and the value associated with the geometric midpoint between the pair of points. This process is then repeated for all initial recording sites (*i*) and the final RVI value plotted over the domain as the mean of all values associated with the same location. A colour map is then used to highlight small or negative RVI values to reveal regions most susceptible to reentry. For more information, see Child *et al* [10].

#### 2D versus 3D Computation

In the 2D model, all data within a circle of radius *r*_*s*_ around each site was used in the computation of the RVI. In the two 3D models, however, either data only from the recording surface could be used, in a similar manner to the 2D model calculation, or alternatively, full 3D data regarding ATs and RTs within a spherical region (radius *r*_*s*_) around each site could be used. In this study, we quantify both the overall minimum value of RVI located within the domain (*RV I*_*min*_) as well as the percentage of the spatial RVI map produced (surface or volume) having values < 50 ms (*RV I*_%<50*ms*_).

#### Stimulation Protocols

**Fig 2:**
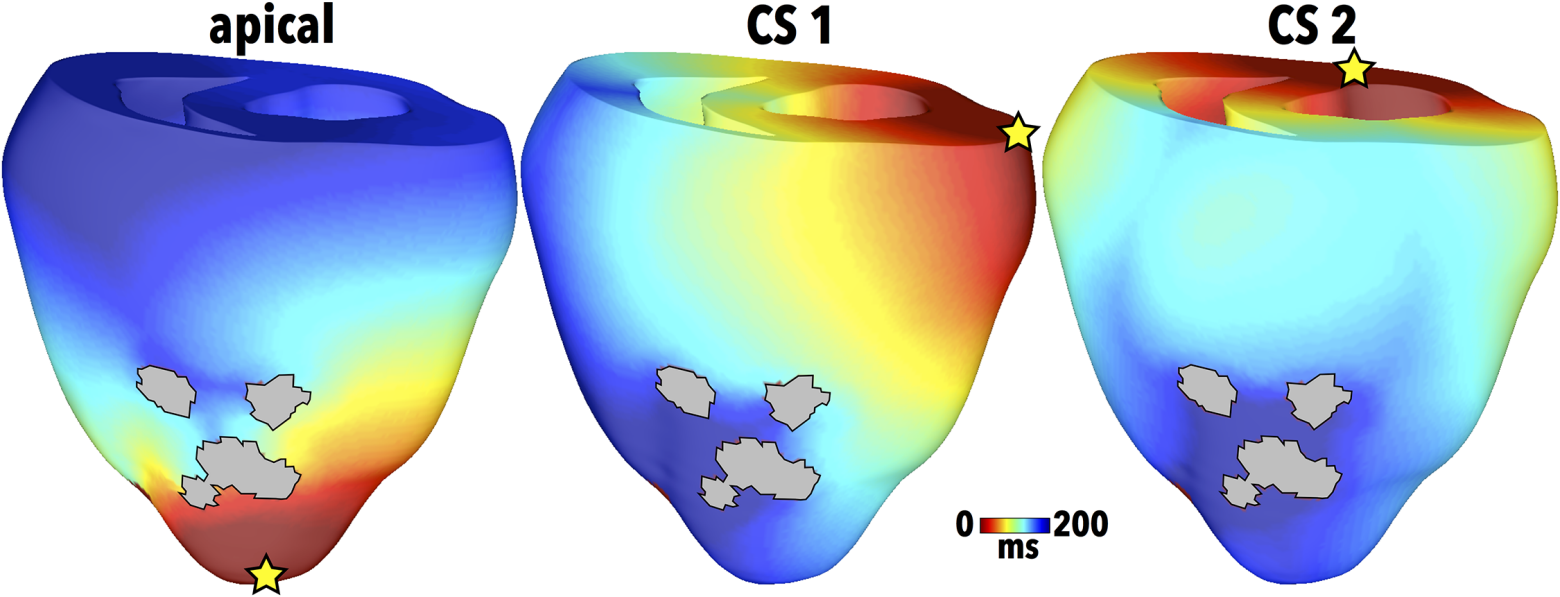
Pacing locations in the rabbit ventricular model (for the LAD scar anatomy) signified by yellow stars with corresponding S1 AT maps for apical (left), CS1 (centre) and CS2 (right) sites.

All RVI calculations were performed on the S2 ATs and RTs following S1S2 stimulation. In some cases, the S2 stimulus also induced reentry. Unless otherwise specified, an S1S2 coupling interval (CI) of 300 ms was used and the APD of the distal tissue was 300 ms. In the 2D sheet model, the S1S2 pacing was applied from a point in the upper left quadrant. In the 3D cuboid model, pacing was applied along the *x* = 0 surface of the model. In the rabbit whole ventricular model, the primary pacing location at which both S1 and S2 stimuli were delivered was the apex, as shown in Fig 2 (left). Two additional pacing sites representing access of the catheter via the coronary sinus (CS) were also investigated, shown as CS1 (centre) and CS2 (right) in Fig 2.

### Simulating Ablation Lesions

In the clinical setting, ablation lesions penetrate approximately 1 cm in depth [27] with the goal of making them close to completely transmural. In our rabbit ventricular model, ablation lesions were created with radii between 3–4 mm to ensure similar lesion penetration through the ventricular wall. Tissue residing within the lesion was assigned properties of insulating connective tissue, similar to the necrotic scar.

## Results

### Basic Use of the RVI Algorithm and Parameter Sensitivity

In this Section, the RVI algorithm is applied to the idealised 2D model with a default search radius of 2.5 mm and recording resolution of 1 mm, unless specified otherwise. Note that the data, and mesh files, corresponding to the fundamental results presented in this Section are made available in the Supporting Information file S1 Data.

#### The RVI Algorithm

**Fig 3:**
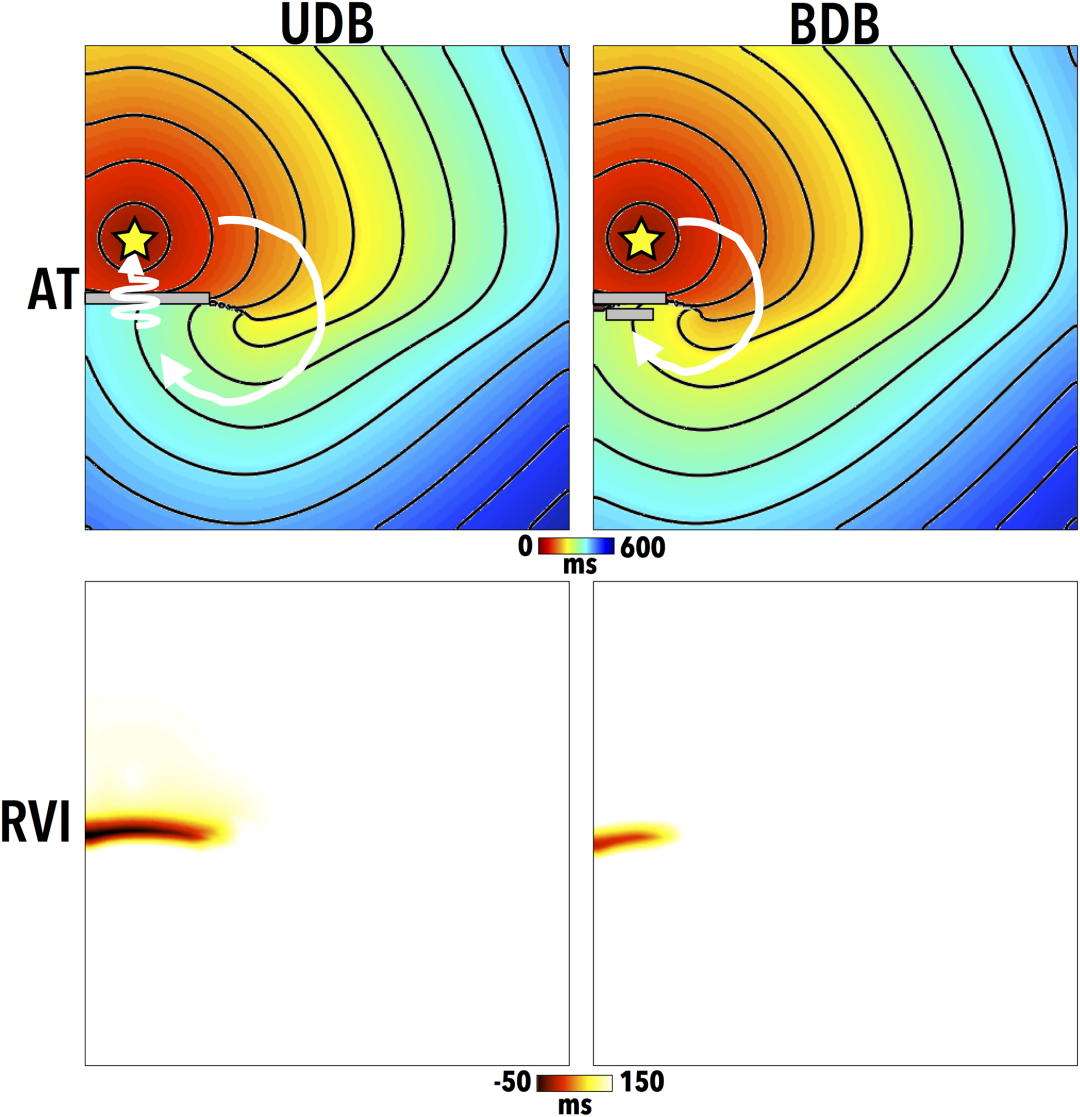
AT maps (top) for the premature S2 beat, along with computed RVI maps (bottom) for cases of both uni-directional block (UDB) and subsequent successful reentry (left) and bi-directional block (BDB) and failed reentry (right). Grey lines highlight approximate lines of initial conduction block, with white arrows highlighting wavefront paths. Pacing locations are shown by a yellow star in the AT maps.

Initially, we demonstrate the basic use of the RVI algorithm in both a situation of induced reentry and failed reentry in which only the dynamic electrophysiological properties of the tissue are altered between the two cases (specifically, the APD of the distal tissue) to produce different induced outcomes, as originially shown in Child et al (2015) [10]. Fig 3 shows the AT maps (top) following the S2 stimulus for the two different simulated episodes. In the left case, uni-directional block (UDB, reentry) occurs. Here, the S2 wavefront is initially blocked from entering the lower distal tissue (with lengthened APD of 309 ms) as it remains refractory, and is thus forced to propagate around the line of block (to the right), highlighted by a grey line in Fig 3. The distal region subsequently recovers, allowing the wavefront to propagate downwards, pivoting around the edge of the line of block in a clockwise manner, highlighted by the white arrow. Here, the long path taken by the S2 wavefront provides sufficient time for the proximal tissue to recover, meaning the wavefront is then able to successfully propagate back across the line of initial block and reenter. In the right case, bi-directional block (BDB, failed reentry) occurs. Here, the distal tissue is assigned a shorter APD (301 ms) meaning that the line of block is shorter as the initially blocked wavefront can propagate earlier into the recovered distal tissue. In this case, the proximal tissue remains refractory when the wavefront attempts to reenter into it, and is subsequently blocked.

Fig 3 also shows corresponding RVI maps (bottom) for both the UDB and BDB cases. In this initial case, a search radius of 5 mm and resolution of 200 *μ*m was used. In the UDB case, a large region of low RVI (≲ 50 ms) is seen at the site of the initial reentry. Interestingly, a region of lower RVI is also seen in the BDB case in the same spatial location as the UDB case, despite the failure to actually reenter, albeit with a lower magnitude; this highlights the utility of the RVI metric to identify regions susceptible to reentry, without reentry actually needing to be induced.

#### Dependence of RVI Upon Algorithm Parameters

**Fig 4:**
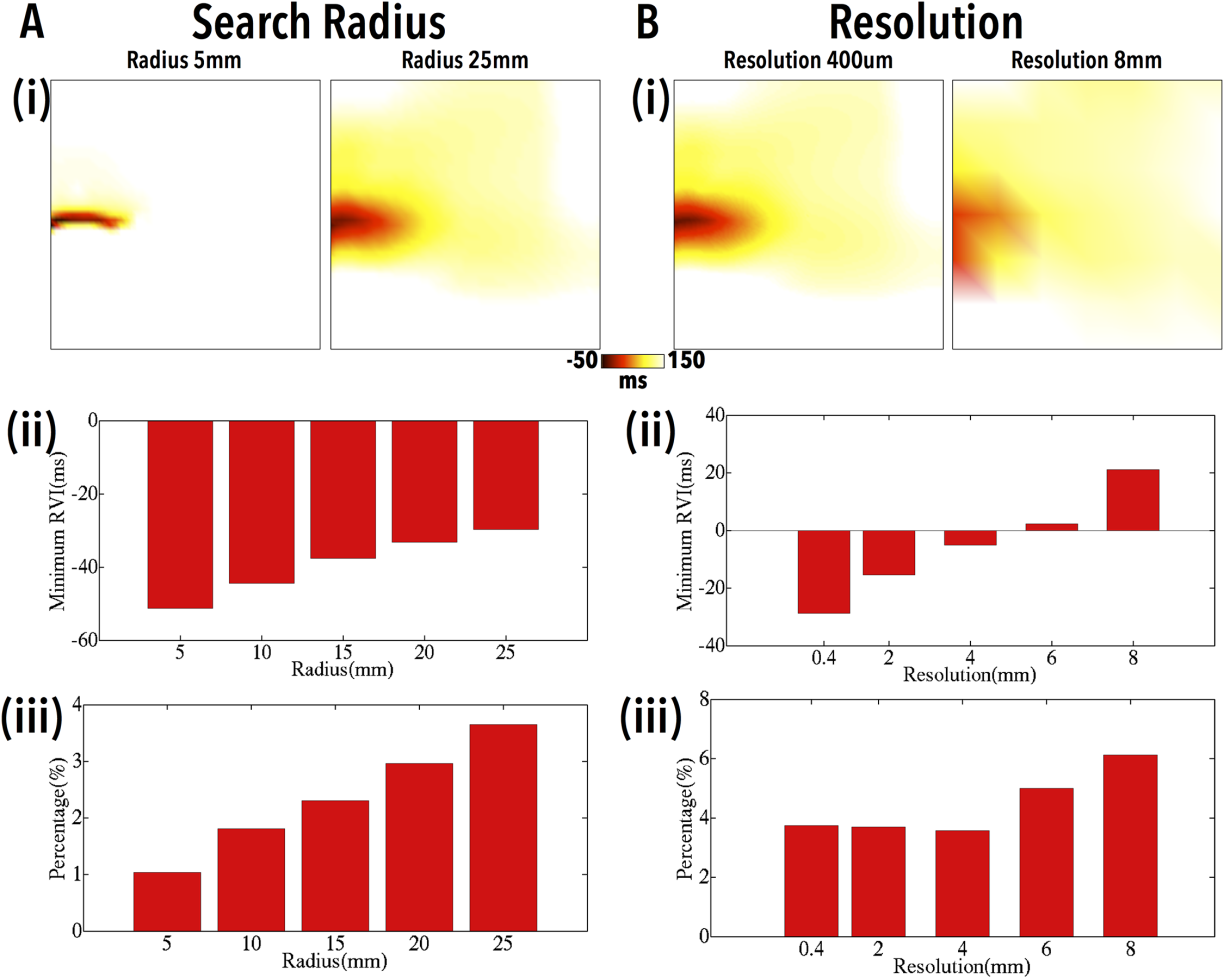
Variation in the RVI as search radius (A) and recording electrode resolution (B) is varied. Example spatial RVI maps (i) are shown for two selected radii (5, 25 mm) and resolutions (0.4, 8 mm), respectively, along with the variation in *RV I*_*min*_ (ii) and *RV I*_%<50_ (iii) as a function of search radius and electrode resolution, respectively.

We now examine how the numerical value of the RVI and its spatial distribution may vary depending upon the parameters defined in the algorithm computation using the same example of UBD shown in Fig 3 above. Fig 4 shows how the overall minimum RVI (panels (ii)) as well as the percentage of tissue with RVI < 50 ms (panels (iii)) changes as the search radius (A) and the electrode resolution (B) are varied; in each case, example RVI maps are shown at the extreme values of radius and resolution (panels (i)), respectively. In the case of varying search radius (where a fixed resolution of 1 mm is used), although a monotonically less negative value of RVI is seen as search radius increases, a more widely distributed region of relatively low RVI (< 50 ms) is also seen. Thus, as evidenced in the RVI maps (Fig 4a(i)), for all radii examined, the algorithm successfully identifies the region of low RVI at the site of reentry.

In the case of varying recording electrode resolution (where a fixed search radius of 25 mm is used), the RVI maps of Fig 4B(i) show that the overall spatial region of reduced RVI remains relatively constant as electrode resolution is reduced. However, the minimum values of RVI within this region become less negative as resolution is increased. Importantly, even for low spatial resolutions similar to those used in the clinic (∼8 mm), the region of low RVI can still be clearly delineated.

#### Dependence of RVI on Arrhythmogenicity of Substrate

**Fig 5:**
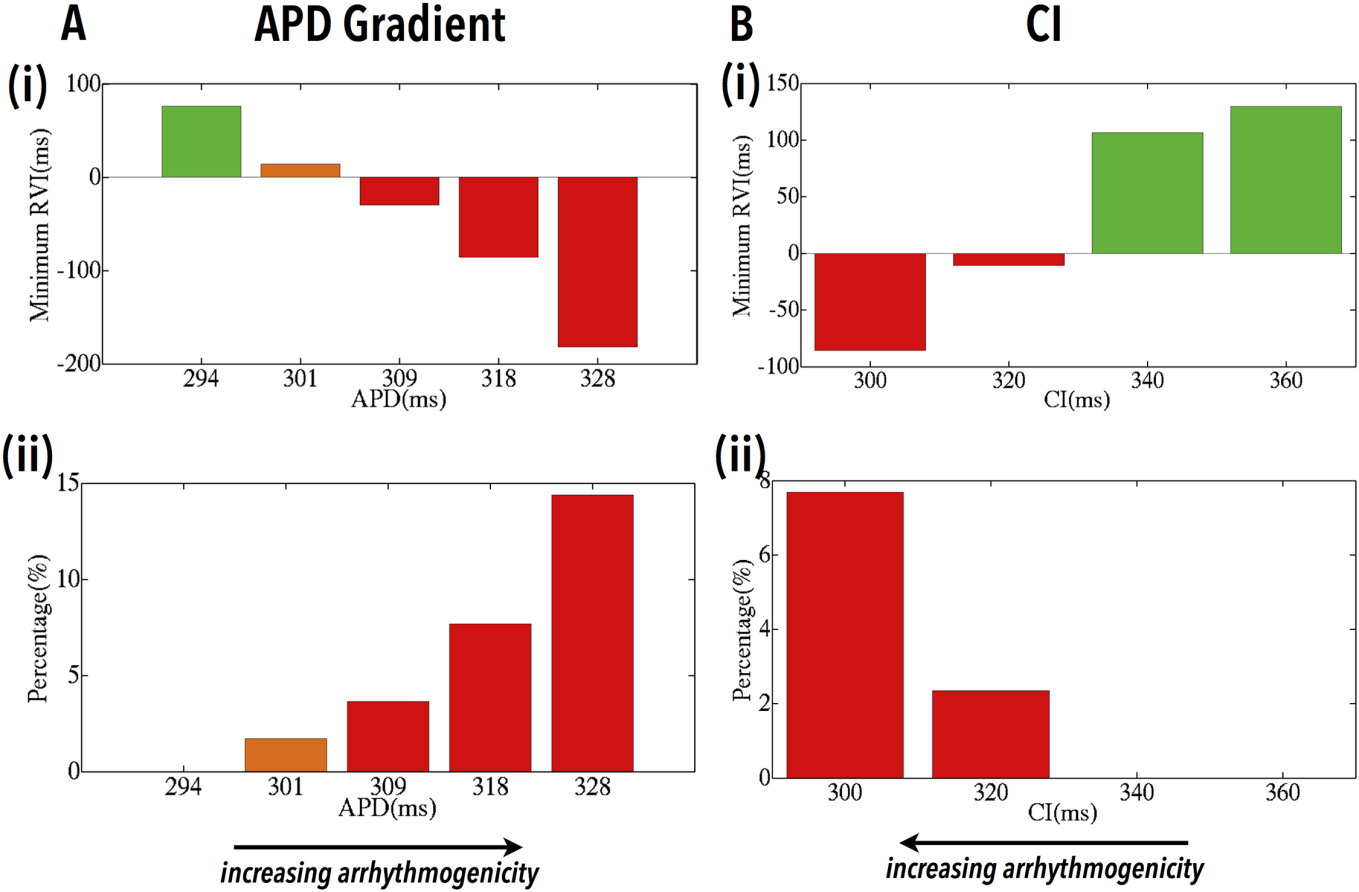
Dependence of RVI on the arrhythmogenic potential of the substrate. Variations in *RV I*_*min*_ (i) and *RV I*_%<50_ (ii) as a function of the APD of the distal region (A) and the S1S2 CI (B). Bars are colour-coded as induced UDB/reentry (red), BDB/failed reentry (orange) and no block/propagation (green).

During a reentry induction protocol such as that used in the sections above, it is known that increasing the APD gradient between disparate regions, or the use of shorter S1S2 CIs, facilitates the induction of reentry [11]. In Fig 5A we show how the computed RVI varies during different induction protocols in which the APD of the lower distal region is varied (with default CI of 300 ms). For higher APDs (> 309 ms) UDB successfully occurs, whereas for lower APDs, either BDB or no block at all occurs. Furthermore, the higher the APD of the distal region (and thus the larger the APD gradient between proximal and distal regions), the RVI is able to more robustly identify the region of reentry through a lower value of *RV I*_*min*_ and a higher value of *RV I*_%<50_, highlighting the increased arrhythmogenic potential of the substrate.

Fig 5B shows how the computed RVI varies for different S1S2 CIs. Here, as the tissue is paced more prematurely (lower CI), UDB and reentry induction becomes more likely, whereas for less premature stimuli, no block at all occurs. Furthermore, for shorter CIs, again, the RVI successfully identifies the increased arrhythmogenic potential of the substrate through lower values of *RV I*_*min*_ and higher values of *RV I*_%<50_.

### Detecting Intramural Scar-Related Reentry

Having demonstrated the utility of the RVI metric to successfully identify sites of reentry (as well as sites vulnerable to reentry) using idealised 2D *in silico* models, we now examine its utility in the case of infarct scar-related reentry in addition to the effects of intramural scar in a higher dimensional (3D) model. In this case, a search radius of 5 mm and an electrode resolution of 0.2 mm (corresponding to the discretisation of the mesh) was used.

**Fig 6:**
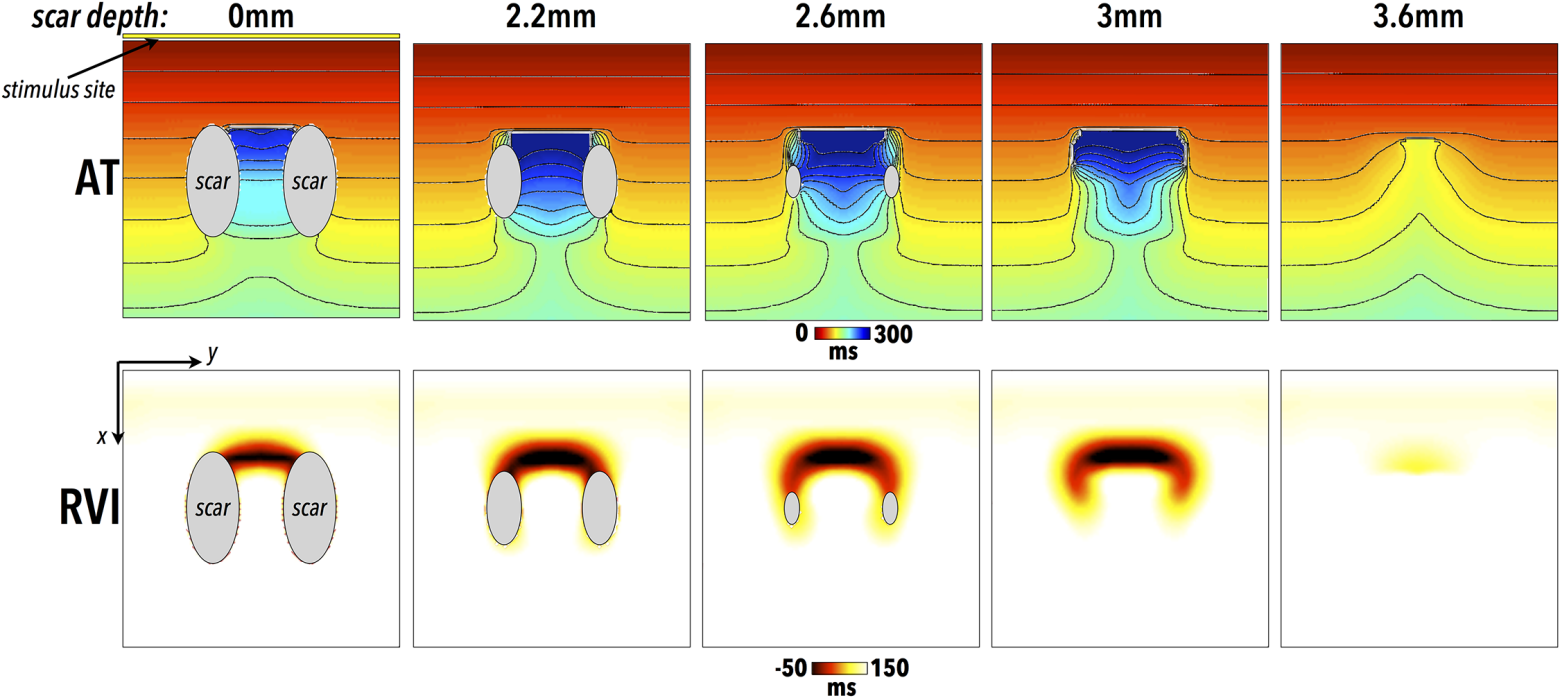
Location of scar-related reentry by RVI metric and dependence upon scar depth. All data is shown on upper (*z* = 0) surface of cuboid meshes, with the scar regions located at progressively deeper depths from left-to-right (and are thus not visible on surface for depths of 3.0 and 3.6 mm as are wholly intramural). Surface AT maps (top) for the premature S2 beat, in addition to corresponding RVI (bottom) maps, in the case of scars at different depths beneath the recording surface. Regions of necrotic scar on the tissue surface are shown in grey. Pacing stimuli are applied to the *x* = 0 surface, shown in first panel by yellow line.

Fig 6 shows AT and RVI maps in cases of reentry induction within the 3D cuboid scar models shown in Fig 1, with each column showing maps corresponding to scars at different depths beneath the surface. In all cases, activation of the S2 wavefront is blocked at the mouth of the isthmus close to the pacing site as the tissue here has yet to recover. Activation does, however, successfully propagate around either side of the scar and enters the scar isthmus at the far end. In all cases simulated, the wavefront subsequently exits out of the isthmus to re-excite the tissue proximal to the pacing site, thus reentering [28]. Importantly, for all scar depths a region of low RVI is located at the exit site of the isthmus; even for entirely intramural scar, the effects upon the AT and RT on the recording surface are sufficient to still result in an appreciable region of low RVI, highlighting the site of reentry. Here, activation is slightly slowed on the surface due to propagation through BZ tissue. However, it is the conduction block that occurs in the tissue depth that acts to reduce APD of the surface tissue through electrotonic interactions and which primarily drives the reduction in RVI.

### Detecting Scar-Related Reentry in a Whole Ventricular Model

Patients presenting with recurrent scar-related VT requiring ablation often have complex infarct scar anatomies. In order to assess the ability of the RVI approach to identify critical reentry sites in the context of such complex scars, the algorithm is now applied to the whole ventricular model with LAD scar anatomy, shown in Fig 1. Here, the search radius used in the RVI calculation was 5 mm, approximately scaled in comparison to that used in the human ventricle [10], with an electrode resolution set to the corresponding discretisation of the finite element mesh. In the first instance, apical pacing for the S1S2 protocol is used, as performed in the previous clinical study [10], with an S2 CI of 220 ms. In this case, the S2 wavefront blocks at the BZ on the proximal side of the scar, forcing it around the scar edges and entering into the network of isthmuses from the distal side, similar to the dynamics seen above in the more simplified scar case (Fig 6). A complex sustained reentry then ensues following the S2 beat which lasts for the duration of the simulation (approximately two and a half reentry cycles).

**Fig 7:**
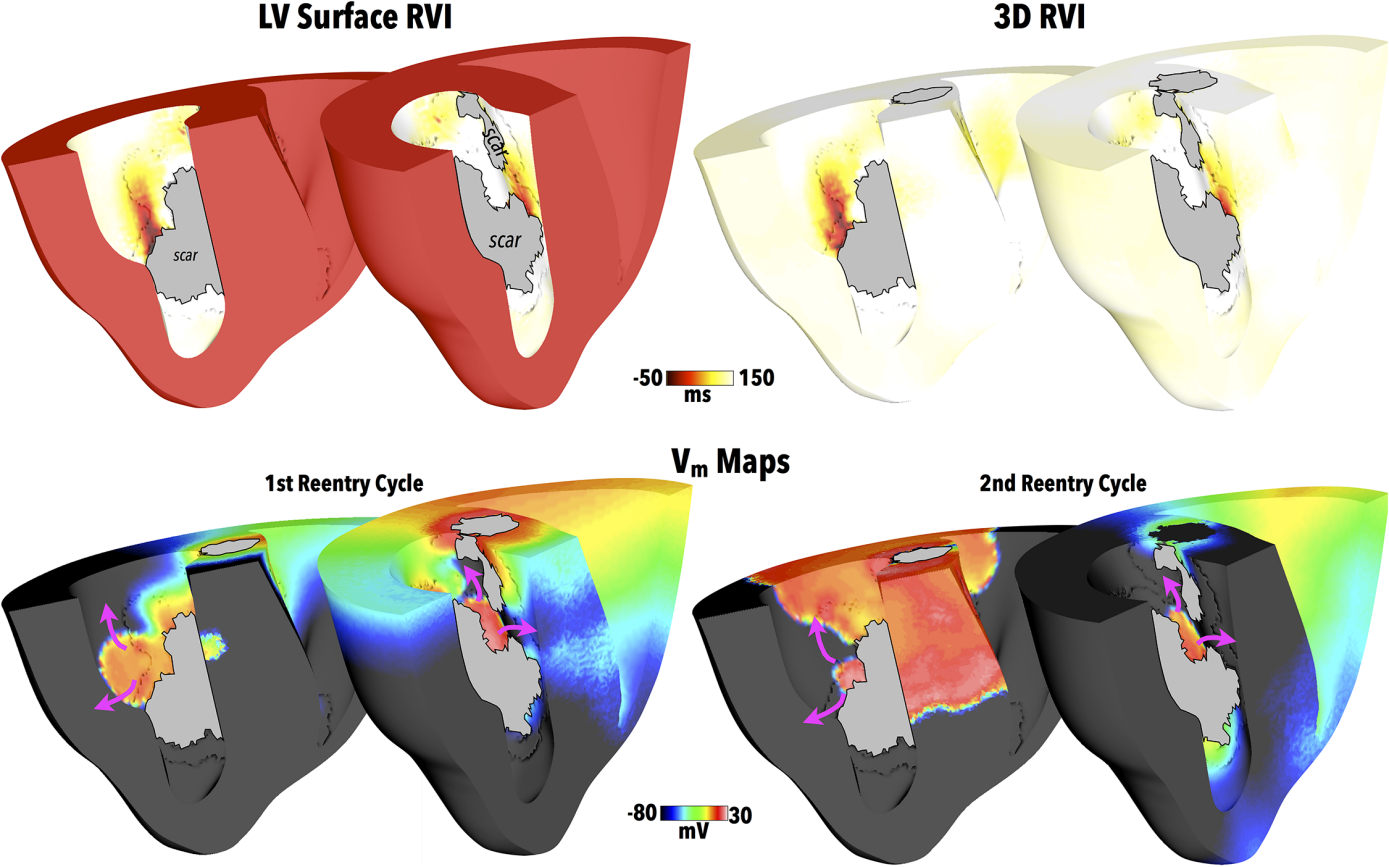
RVI following apical pacing with LAD scar. Upper panels show RVI values computed on LV endocardial surface (left) and on all 3D myocardium (right) for the LAD scar following apical pacing with clipping planes used in 2 views to facilitate visualisation of endocardial surfaces. Greyed regions signify necrotic scar on endocardial surface. Snap-shots of *V*_*m*_ distributions during the first two cycles of reentry are shown in the lower panels, highlighting exit sites of induced reentry with purple arrows used to show reentrant activation paths.

Fig 7 shows the RVI computed both using only data from the LV endocardial surface as well as using all of the 3D intramural data available within the model. Comparing the RVI maps on the LV endocardium, little difference is seen either qualitatively or quantitatively between the surface-only or full 3D computation methods. Furthermore, as seen in the Figure, both RVI maps clearly show low RVI regions which correspond to exit sites associated with the reentrant wavefront dynamics around the scar, highlighted in Fig 7 (lower panels), where both the first and second reentrant cycles are shown.

### Simulation of RVI-Guided Ablation

**Fig 8:**
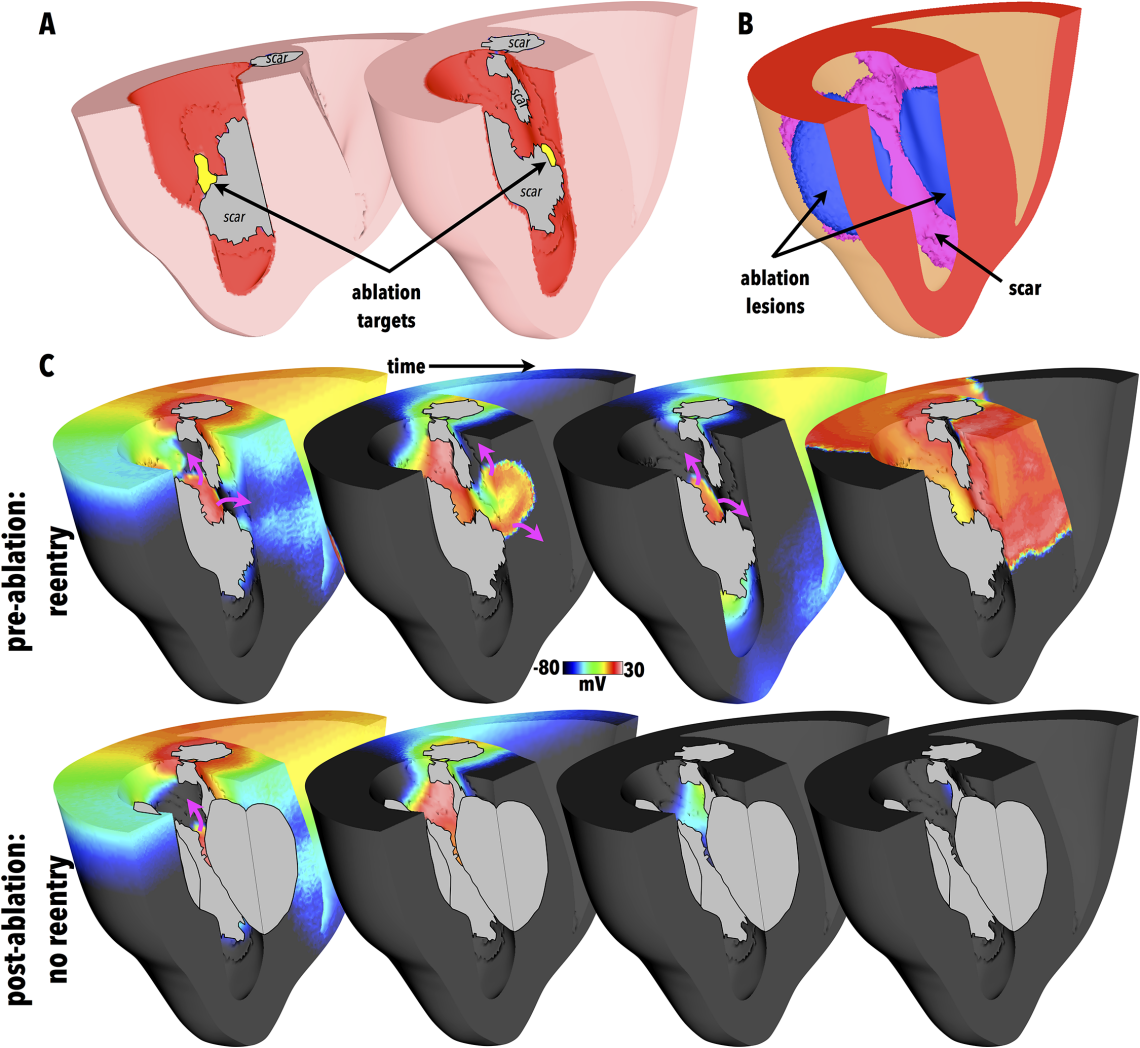
(A) Target ablation locations identified as regions with LV surface RVI < 25 ms, highlighted in yellow, with clipping planes used in 2 views to facilitate visualisation of endocardial surfaces. Greyed regions signify necrotic scar on endocardial surface. (B) Visualisation of corresponding ablation lesion (blue) in addition to scar (purple). Opaque surfaces used to facilitate intramural visualisation. (C) Comparison between activation dynamics pre- (top) and post- (bottom) ablation showing snap-shots of *V*_*m*_ distributions. Purple arrows show wavefront propagation directions. Greyed regions signify necrotic scar as well as lesion.

Of key clinical importance is whether the regions of low RVI identified in the maps correspond to critical target sites for catheter ablation that may successfully terminate, or prevent, the reentry. Consequently, the LV surface RVI map from Fig 7 was used to identify target ablation sites as those surface regions with RVI < 25 ms; such target regions are identified in Fig 8A. Simulated ablation was performed from these target regions as described in the Methods, with Fig 8B showing the resulting lesions formed.

In the Section above, reentry ensued following the S1S2 pacing protocol used to compute the RVI metric. Thus, to assess the success of the simulated ablation, the exact S1S2 apical pacing protocol which successfully induced sustained reentry in Fig 7 was repeated in the newly ablated model, administered from the same pacing location (apex).

In this case, no reentry could be initiated and the activation died away. Fig 8C compares the wavefront dynamics between the successful reentry induction of the model pre-ablation, with the failed reentry induction of the post-ablation model, demonstrating how the formation of the ablation lesion successfully prevents reentry occurring.

### Application to Other Scar Morphologies

**Fig 9:**
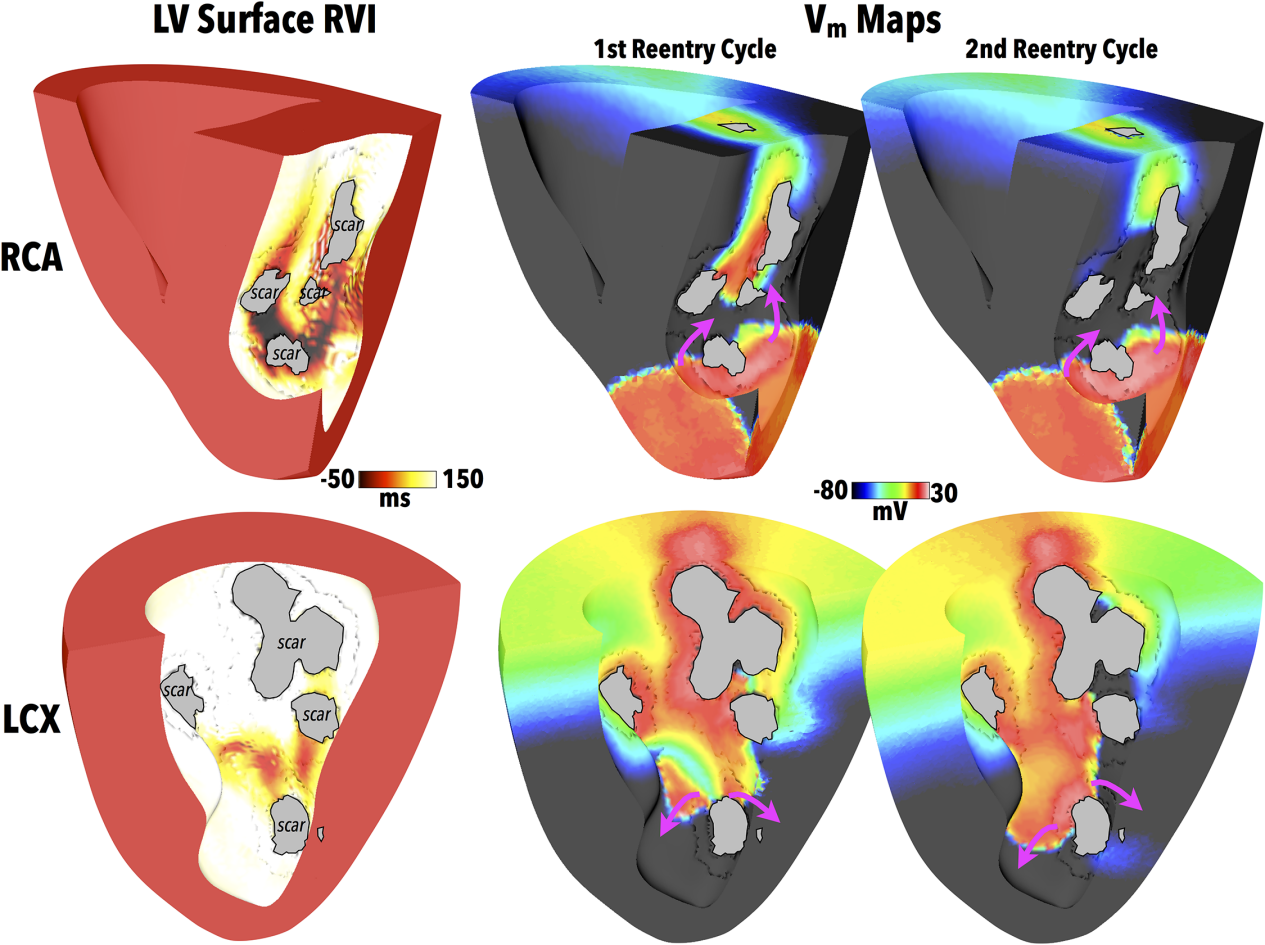
RVI within different scar anatomies. RVI computed on LV endocardial surface (left) for both the RCA and LCX scar models, along with snap-shots of *V*_*m*_ distributions (right) showing exit sites of induced reentries during the first two reentry cycles. Purple arrows show wavefront propagation directions. Greyed regions signify necrotic scar on endocardial surface.

Having demonstrated the ability of the RVI approach to successfully identify critical reentry sites in one single scar anatomy, we now apply the algorithm to two more scar anatomies (shown in Fig 1) located in disparate regions within the ventricle and each with significantly different scar/BZ structures. Fig 9 (left) now compares RVI maps computed from similar apical S1S2 pacing protocols, but in this case applied to the RCA and LCX scar anatomy models. As in the LAD scar model of Fig 7, an S2 CI of 220 ms was used resulting in initial block of the S2 wavefront at the proximal side of the scar, followed by ensuing sustained reentry following the S2 beat.

As before, regions of low RVI (computed on the LV endocardial surface) are seen to correspond to exit sites of activation wavefronts in the scar during the reentry shown by the corresponding *V*_*m*_ distributions in Fig 9 (right). Importantly, these regions of low RVI were then used to identify target sites on the LV endocardial surface for the generation of ablation lesions, using the same method as used in the LAD scar model. Following lesion creation, identical S1S2 apical pacing protocols were administered to the RCA and LCX models from the same apical site used to compute the RVI and which successfully induced reentry pre-ablation. In contrast to the cases pre-ablation (Fig 9 (right)), no reentry could be initiated, and all activation died away following the S2 beat, in a similar manner to the LAD scar model of Fig 8(c).

### Utility of Alternative Pacing Locations

**Fig 10:**
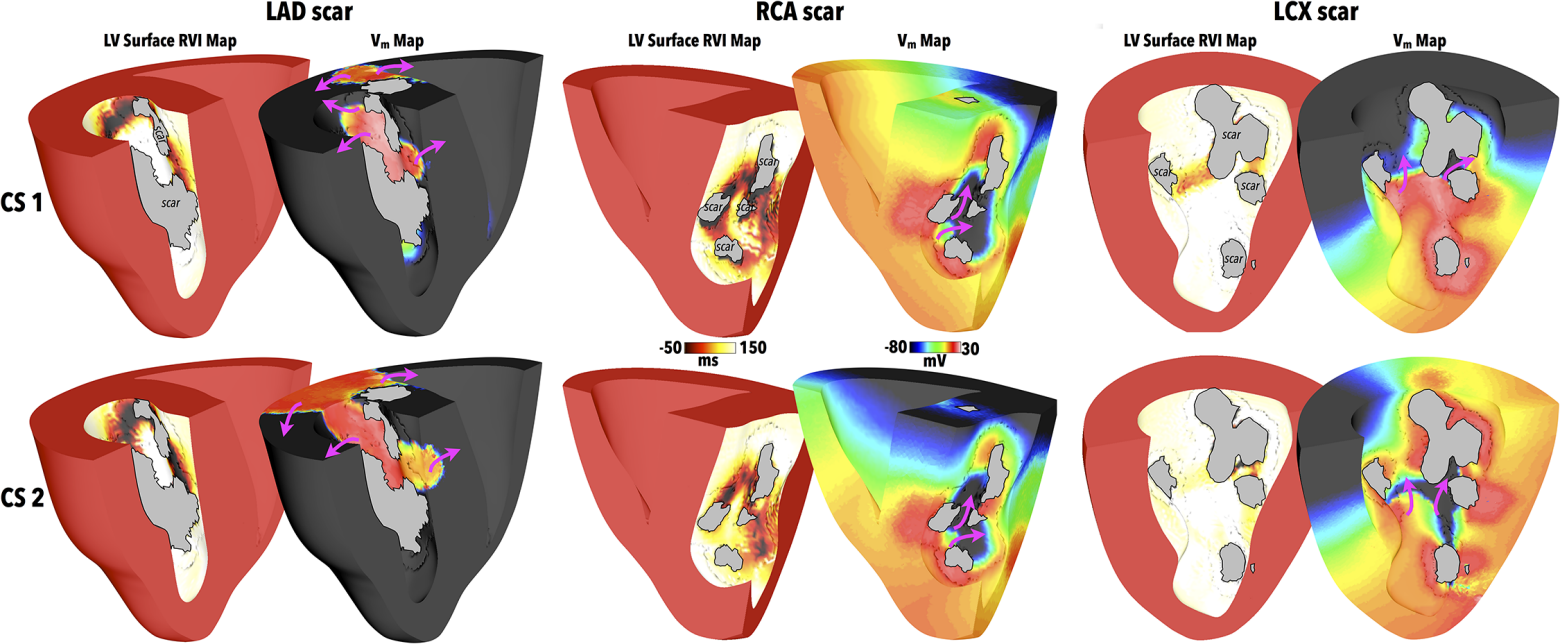
RVI using CS pacing locations (highlighted in Fig 2) for different scar anatomies. RVI computed on LV endocardial surface along with snap-shot of *V*_*m*_ distribution showing exit sites of induced reentries for the LAD (left), RCA (centre) and LCX (right) scar models following pacing in CS1 (top panels) and CS2 (lower panels) locations. Purple arrows show wavefront propagation directions. Greyed regions signify necrotic scar on endocardial surface.

As is common in the case of recurrent scar-related VT, complex scar anatomies may support a number of different reentrant circuits with a corresponding number of isthmus exit sites that ideally need to be targeted to prevent re-occurrence. In the case of such complex scars, the specific direction and location at which the paced wavefront interacts with the scar *may* be critical in determining its subsequent pathway through the scar and thus the reentrant circuit(s) and exit site(s) identified by the RVI approach. Here, we therefore assess the potential utility of different pacing locations from which to perform the RVI protocol. As in the case of apical pacing, pacing from both of the CS locations also induced reentry following the S2 beat in all scar models. However, due to the different direction of wavefront interaction with the scars, reentrant dynamics were different for different pacing locations (to different degrees depending on the scar). Fig 10 shows RVI maps for all three scar anatomies following pacing at the two different CS locations delineated in Fig 2, along with the corresponding *V*_*m*_ images which highlight wavefront pathways through the scars.

In the case of the LAD scar, the *V*_*m*_ images suggest similar wavefront patterns through the scar following pacing from both CS locations, in comparison to when pacing was performed from the apex (Fig 7). Consequently, the corresponding RVI maps of Fig 10 highlight similar regions of low RVI, both between the two different CS pacing locations, and in comparison to the RVI map following apical pacing in Fig 7.

In the RCA case, the highlighted regions of low RVI in Fig 10 appear similar between both CS pacing protocols, underscored by the similarity in the corresponding wavefront patterns. When compared to the RVI map following apical pacing (Fig 9), although this also shows large, distributed regions of low RVI, important differences exist. Examining the RVI maps more closely reveals that the apical protocol highlights more clearly low RVI areas at exit sites in the scar nearer the apex of the ventricles, whereas the CS protocols seem to highlight better low RVI areas at exit sites nearer to the base.

Finally, in the case of the LCX scar, pacing from different locations on the CS produces similar wavefront patterns through the scar, as shown in Fig 10. The computed RVI maps both highlight exit sites higher-up the scar towards the base, although the numerical values of RVI following CS1 pacing highlight these more clearly than following CS2 pacing. However, in comparison to pacing from the apex (Fig 9), both wavefront patterns through the scar and corresponding regions of low RVI are very different to following CS pacing. In the apical protocol, exit sites towards the apex of the scar were delineated as having low RVI, compared to near the base in CS protocols. In addition, it is further noted that the numerical values of the regions of low RVI in the LCX scar model were consistently much higher than both the RCA and LAD RVI values, for all three pacing locations.

Finally, we investigated the robustness of the RVI at identifying ablation targets from a particular pacing location against preventing reentry induction from a different pacing location. Each model had ablation lesions created, guided by the low RVI values following apical pacing (as described in Methods). The same S1S2 pacing protocols were then conducted from the CS1 and CS2 locations, as performed above. Pacing from the CS1 location or the CS2 location failed to induce reentry in both the RCA and LAD scar models. This is in-line with the fact that low RVI regions were in similar spatial locations in the models for all pacing locations considered. However, pacing from both the CS1 and CS2 locations successfully induced reentry in the LCX scar model. Again, this is in-line with the fact that regions of low RVI were located in very different spatial locations when pacing was performed from the apex, compared to the CS locations.

## Discussion

In this study, we have used a series of different computational models to conduct a detailed further investigation into the use of the RVI algorithm to identify sites susceptible to reentry in order to guide clinical catheter ablation. Within idealised models, we demonstrated the importance of optimising the parameters utilised in the RVI calculation, noting that the use of higher resolution data sampling and smaller radius sizes to determine calculation points improves the ability of the algorithm to locate vulnerable regions. Nonetheless, the algorithm still performs well under clinically-relevant conditions. When applied to pathological cases containing realistic infarct scar regions, the RVI algorithm successfully identified exit sites of the reentrant circuit, even in scenarios where the scar was wholly intramural. Within whole ventricle models with highly complex infarct scar anatomies and multiple reentrant pathways, the exit sites identified by the algorithm were seen to be dependent upon the specific location of the pacing stimuli used to compute the RVI metric. However, importantly, simulated ablation of the sites identified as having low RVI successfully prevented the reentry being subsequently initiated from the same pacing location as used in the RVI calculation.

### Dependence of the RVI Upon Parameters Used in its Calculation

Sampling of AT and RT data in the clinic is typically done with decapolar catheters, the resolution of which varies due to the manual manipulation of the catheters. Here, we demonstrate that even resolutions of up to 8 mm between recording sites still produce a qualitatively similar spatial distribution of RVI to that of higher resolutions (Fig 4B). Quantitatively, although the overall minimum value of RVI increases as resolution becomes more coarse, the area of tissue with low RVI (< 50 ms) increases, meaning locating areas of low RVI becomes less specific.

Another variable used in the calculation of the RVI, the search radius *r*_*s*_, is likewise constrained in the clinic, due to the relatively restricted amount of data recorded and used in analysis. We found that increasing *r*_*s*_ produced a more widely distributed region of lower RVI values (higher *RV I*_%<50_), which were visibly less localised (Fig 4A); however, even for the largest *r*_*s*_ considered (25 mm), the minimum value of RVI within the region was still appreciably negative. This has important clinical implications with respect to using the metric to identify the smallest possible region to ablate in order to successfully terminate the VT.

### Prediction of Most Vulnerable Sites

An important requirement of the RVI algorithm is that, as a region of tissue becomes more likely to sustain a reentrant arrhythmia, so it should be highlighted to a greater degree by a lower value of the metric. Due to the restitution property of cardiac tissue, as the S1S2 CI becomes shorter, the APD of the proximal tissue shortens. The proximal tissue therefore repolarises earlier, facilitating reentry by allowing the wavefront to re-excite it after travelling around the line of block. This causes more negative values of RVI to be produced, as RT *decreases*. The RVI value is further decreased in this region, due to the concurrent effect of CV restitution, meaning that the initially blocked wavefront travels more slowly around the line of block, attempting to reenter across it at a later time (thus *increasing* AT in the RVI calculation). This explains why the slope of the restitution curve is important for the induction of reentry, and while a steep slope tends to promote reentry [29], inducibility is not dependent on an absolute value of the maximum slope (see also [11]). Similar arguments can be formed to explain the increased likelihood of sustained arrhythmia due to a higher degree of APD heterogeneity between proximal and distal regions due to the initially blocked wavefront being forced further around the line of block, providing longer for the proximal tissue to recover. Again, AT of the neighbouring distal tissue is increased, lowering the computed RVI. In both cases, as shown in Fig 5, the increased vulnerability to reentrant circuit formation is highlighted by both lower overall RVI values and an increased spatial region of low RVI. However, we note that both CI and APD represent a ‘window of vulnerability’. If CI is too low, capture of the pacing stimulus in the proximal tissue fails; if it is too long, the APD heterogeneity between tissue types is not exploited and no initial block occurs. The APD of the distal tissue needs to be longer than that of the proximal tissue in order to cause uni-directional block of the premature beat, yet if it becomes too long, it will adversely increase activation wavelength such that reentry cannot be contained within the circuit [30].

These effects of restitution also play a key role in one of the great strengths of the RVI approach: the fact that sites vulnerable to reentry may be identified without reentry actually needing to be induced [10–12]. As evident in Fig 3, even in the case of BDB the same vulnerable region was identified as having low RVI, compared to the case in which reentry did occur (in this case following a change in assigned APD difference between proximal-distal areas). In the whole-ventricular models, full reentry was initially induced via the S1S2 protocols applied to the tissue and used to compute the RVI; this was done so as to verify arrhythmia termination following simulated ablation. In the case of anatomical reentry, the physical path-length remains the same; however, as described above, differences in APD and CV induced by restitution effects can correspondingly alter the wavelength and thus dictate the success of reentry. In the clinic, S1S2 intervals can easily be lengthened, preventing reentry occurrence, but it is still possible to highlight vulnerable regions (through a low RVI value) which may facilitate reentry under different electrophysiological conditions.

### Identifying Intramural Reentry

An important question originating from the initial study by Child et al [10] was the ability of the metric to successfully identify regions of reentry from the surface under circumstances where the reentry itself (and consequently the scar-related pathway or isthmus) was entirely intramural. Although such an issue is present as a potential problem in any kind of mapping and ablation technique performed from the surface, here we demonstrate the strength of the RVI to successfully identify such intramural reentry sites, as shown in Fig 6. Of prime significance in this respect, is the fact that the RVI also incorporates information regarding localised repolarisation times from the surface. During repolarisation, electrotonic effects—acting over spatial length-scales of a few length constants [31]—have the effect of spatially-smoothing differences in RT such that regions of heterogeneous repolarisation, albeit beneath the recording surface, may be transduced through the surface by electrotonic interactions. Thus, even when data is recorded only from the surface and used to compute the RVI, these important differences are picked-up by the algorithm and successfully contribute to highlighting regions susceptible to reentry.

### Practical Use of RVI in Complex Scar Geometries

The majority of patients presenting with chronic scar-related VT tend to have complex scar morphologies with a number of different possible surviving pathways that may support reentrant circuits and thus necessarily multiple possible exit sites that may need to be targeted. Here, we have demonstrated that the RVI algorithm can successfully identify the correct exit sites corresponding to the reentrant circuit induced during the S1S2 induction pacing protocol. However, importantly, we have shown that, in the case of complex scars, changing the location of the pacing site from where the RVI protocol is performed, may, in some cases, significantly shift the exit sites identified by the RVI (as witnessed in the LCX model—Figs 9 and 10); however, in other scar morphologies, despite shifting the pacing site from apex to the CS, the exit sites highlighted by the RVI remain in similar spatial locations (as witnessed in the LAD model—Figs 7 and 10), although the numerical values and exact distributions were seen to change. In these cases, as the pacing location is altered, the initial interaction site of the wavefront with the scar will also be different, thus experiencing block (by the region of prolonged APD at the BZ) at a different exit site. Consequently, the site at which the blocked wavefront will then enter into the scar (to traverse it via an isthmus) will be correspondingly different, as will the resulting point at which it attempts to exit. Thus, if some form of knowledge regarding the anatomy and/or location of the scar (from MR, for example) is known prior to ablation, it may therefore be possible to select the optimal pacing site from which to perform the RVI protocols in order to identify the most probable critical exit site.

Performing simulated ablation using specific target sites identified by thresholding the RVI maps were successful in preventing the induced reentry when the same S1S2 induction protocol (which induced a reentrant circuit pre-ablation) was re-applied. Here, we note that testing for reentry induction was initially only performed at the same site at which the RVI pacing protocol was performed to identify the corresponding ablation targets. The purpose of this part of the study was a proof of concept to demonstrate the specificity of the RVI in identifying potentially critical ablation sites in complex scar anatomies. Although our ablation lesion was based on those suggested in clinical studies [27], we did not perform a full sensitivity analysis on the size or depth of the defined ablation lesion and note that the ultimate success of such a lesion may depend upon these factors. In the clinic, the goal during ablation is that lesions are created as close to transmural as possible. Moreover, during clinical application of the algorithm it may be that the pacing protocol could be repeated until the region of low RVI was no longer present, with ablation lesions increased in size until this point is reached.

We further emphasise the utility of the RVI metric to identify optimal ablation targets (i.e. those most vulnerable to reentry) over-and-above, for example, simple AT mapping. As demonstrated here and in our previous study [10], the power of the RVI metric lies in the use of localised RT information, thus assessing the potential for the blocked wavefront to actually successfully complete a reentry loop [11, 12]. We further highlight (in Fig 5B) that in the case of RVI mapping, if shorter S1S2 CIs are used, then, due to the above-described effects of restitution, the RVI should highlight increasingly susceptible areas with lower RVI values. However, AT maps would remain similar, still only identifying the same spatial region of block. Related to this, as also described above, as heterogeneity in refractoriness may be transduced through the myocardial wall due to electrotonic interactions in a manner that activation may not, the RVI approach contains important information regarding intramural dynamics that may not be present purely in AT mapping.

As discussed above, although reentry may be prevented by ablating tissue beneath those vulnerable areas identified by low RVI values, for complex scars, it may well be still possible to induce reentry by pacing from different anatomical locations due to the existence of multiple isthmuses and exit sites which may have a critical role in the clinical VT. In this work, we only demonstrated that reentry could not be re-initiated (following ablation) when pacing from the same location at which the initial RVI protocol was performed (and which subsequently initiated reentry pre-ablation). However, for certain complex scar morphologies, we found that reentry could still be successfully initiated if pacing was performed from a different location to that used to compute the RVI to guide the ablation lesion. This was the case for scar models which showed a big shift in low RVI location upon moving the pacing location (LCX scar model). However, for scar models which consistently highlighted exit sites with low RVI at similar locations as the pacing site was moved (RCA, LAD models), reentry was still not possible to induce.

The findings from our study suggest that a combination of different pacing sites may be utilised in order to create multiple RVI maps which may thus identify a number of anatomically-distinct target sites (exits) for catheter ablation. Those targets which are identified from more than one RVI map from different pacing sites may potentially be thought of as the more critical ablation target, which may be most dominant in the clinical VT. Nonetheless, all exit sites which are identified from any of the pacing locations represent important ablation targets, emphasing the ability of the approach to identify multiple potential exit sites (and potentially all exit sites associated with a given scar) which is not possible with other mapping techniques. By identifying a number of critical exit sites through different RVI pacing locations, it may be possible to successfully prevent reentry occurrence by the creation of smaller (more targeted), minimally invasive lesions.

### Study Limitations

Although it has been suggested that lateralisation of connexin distributions occurs within the BZ cells (reducing conduction anisotropy), the presence of fibrosis between muscle bundles and sheets has the converse effect of increasing anisotropy [32]; thus we decided to uniformly reduce conductivity in all directions in order to achieve the effect of reduced CV in the BZ—a prerequisite for sustaining reentry.

The electrophysiological properties of the rabbit are known to be most similar to the human with respect to the dynamics of reentry circuits [30, 33, 34]. The use of a rabbit ventricular model facilitated a detailed computational analysis to be performed with significantly reduced computational burden compared to a human ventricular model. In all cases, regardless of the species, how the reentry was initiated or the nature of the reentrant circuit (anatomical or functional) once induced, the RVI metric successfully identified the vulnerable regions; this is to be expected, as the metric is designed to only assess wavefront-wavetail interactions, independent of their nature [10–12]. We emphasise here that the purpose of this modelling study was not to conduct a detailed investigation into the mechanisms of scar-related reentry and VT. Moreover, for this part of the study, our purpose was to use the models to simulate the ‘phenotype’ of reentry around a scar and then to use the RVI metric to see whether it correctly identified critical sites within the reentrant circuit. Future application of this modelling may be applied to patient-specific clinical models to assess whether any potential differences may arise.

The specific values of the thresholds used to define ATs and RTs in this study may slightly alter the magnitudes of the times computed. This may introduce minor differences in ATs, where the upstroke occurs within 1–2 ms. Altering the definition of RTs from 90% repolarised here (−70 mV) to, for example, 80% repolarised (−60 mV) may shift computed RTs by approximately 10 ms. However, all RTs within the tissue would be approximately shifted by the same amount. As the RVI is a relative metric (i.e. we look for the region of low RVI in relation to the rest of the tissue), such a change is not expected to alter the application of the approach, but may be important to consider when attempting to compare absolute values.

## Conclusions

We have demonstrated that the RVI metric is able to reliably identify pro-arrhythmic exit sites which may be used to guide ablation. We have further shown in idealised 2D models that the metric is robust against the spatial resolution of the clinical recordings and the size of the search radius used in its calculation, whilst in realistic whole ventricular models we have demonstrated it works well in the case of highly complex and intramural scar anatomies. However, our findings suggest that optimal use of the approach involves computing RVI maps from multiple pacing locations.

## Supporting Information

S1 Data The folder S1_Data.zip contains the minimal data underlying the main findings from this study. Specifically, this relates primarily to the data presented in Figs 3, 4 & 5. Data provided constitute AT, RT and RVI datasets, along with corresponding finite element mesh files required for visualisation and analysis.
(ZIP)
