# Supplementary material for: Investigating a Novel Activation-Repolarisation Time Metric to Predict Localised Vulnerability to Reentry Using Computational Modelling
Source: PLoS One. 2016 Mar 2;11(3):e0149342. doi: 10.1371/journal.pone.0149342 (PMC4775046; doi:10.1371/journal.pone.0149342)
Supplement: S1 Data — Specifically, this relates primarily to the data presented in Figs 3, 4 & 5. Data provided constitute AT, RT and RVI datasets, along with corresponding finite element mesh files required for visualisation and analysis. (ZIP) [file pone.0149342.s001.zip › Data/README.pdf]

This folder contains the minimal data underlying the main findings from this study, specifically relating primarily to the data presented in Figures 3, 4 & 5.

Figure 3 data:
This data corresponds to the analysis of the dependence of the RVI in situations of both bi-directional block (BDB) and unidirectional block (UDB). Both activation time (AT), repolarisation time (RT) and RVI data is presented, together with the finite element mesh files necessary for data visualisation.

FFmesh.elem - mesh element file
FFmesh.pts - mesh node file
Fig3_{BDB,UDB}_{AT,RT,RVI}.dat - BDB / UDB cases with AT / RT/ RVI data.

Figure 4 data:
This data corresponds to the analysis of the dependence of the RVI upon both changes in the search radius used in the algorithm and also changes in the resolution of the recording electrodes. AT and RT data is the same as Figure 3 BDB case as only the computational of the RVI is changed here.
Fig4_Rad{5,25}mm_RVI.dat - RVI data for search radii of 5 and 25mm.
Fig4_Res{8mm,400um}_RVI - RVI data for resolutoins of 8mm and 400um.
Mesh_8mm.pts - nodal mesh data required to view Res8mm data above.
Mesh_400um.pts - nodal mesh data required to view Res400um data above.
Mesh_1mm.pts - nodal mesh data required to view Rad5mm and Rad25mm data above.

Figure 5 data:
This data corresponds to analysis of the RVI metric as the arrhythmogenic nature of the substrate is changed, through changes in the APD of the distal tissue and changes in the basic (pacing) cycle length (BCL) of the protocol. Two examples of APDs and two examples of BCLs are shown, with AT, RT and RVI data for all cases.
Fig5_APD{294,328}_{AT,RT,RVI}.dat - AT / RT / RVI data for APDs of 294 / 328ms.
Fig5_BCL{300,360}_{AT,RT,RVI}.dat - AT / RT / RVI data for BCLs of 300 / 360ms.
